# Nutritional and Antioxidant Enhancement of Pasta Enriched with Parota Flour (*Enterolobium cyclocarpum*): A Functional Food Approach

**DOI:** 10.3390/foods14091521

**Published:** 2025-04-26

**Authors:** Víctor Manuel Moo-Huchin, Jorge Carlos Canto-Pinto, Cindialy Yuliet Ku-Canul, Raciel Javier Estrada-León, Alejandro Ortiz-Fernández, Carlos Rolando Ríos-Soberanis, Enrique Sauri-Duch, Félix José Aguilar-Vázquez, Emilio Pérez-Pacheco

**Affiliations:** 1 Tecnológico Nacional de México/IT de Mérida, km 5 Carretera Mérida-Progreso, Mérida C.P. 97118, Yucatán, Mexico; victor.mh@merida.tecnm.mx (V.M.M.-H.); enrique.sd@merida.tecnm.mx (E.S.-D.); felix.av@merida.tecnm.mx (F.J.A.-V.); 2 Tecnológico Nacional de México/ITS de Calkiní, C.A. Bioprocesos. Av. Ah-Canul, Calkiní C.P. 24900, Campeche, Mexico; jccanto@itescam.edu.mx (J.C.C.-P.); 3409@itescam.edu.mx (C.Y.K.-C.); rjestrada@itescam.edu.mx (R.J.E.-L.); aeortiz@itescam.edu.mx (A.O.-F.); 3 Centro de Investigación Científica de Yucatán, A.C. Unidad de Materiales, Calle 43, No. 130 x 32 y 34, Colonia Chuburná de Hidalgo, Mérida C.P. 97205, Yucatán, Mexico; rolando@cicy.mx

**Keywords:** parota flour, functional foods, antioxidant capacity, dietary fiber, protein enrichment, pasta quality

## Abstract

The objective of this research was to evaluate the physicochemical quality, bioactive compound content, and antioxidant capacity of pasta made with durum wheat semolina and partial substitutions of parota flour (*Enterolobium cyclocarpum*). Fettuccine pasta formulations were prepared with different percentages of parota flour (0%, 10%, 30%, and 50%). The methodologies included proximate chemical composition analysis, dietary fiber determination, total phenolic content, antioxidant capacity (ABTS assay), and reducing power, as well as cooking quality tests and color analysis. The results showed that the incorporation of parota flour significantly increased protein content (up to 22.06 g/100 g), total dietary fiber (up to 22.1 g/100 g), and total phenolic compounds (up to 23.35 mg/100 g). Additionally, higher antioxidant capacity and reducing power were observed in the pastas with higher parota flour content. In terms of cooking quality, the parota flour-enriched pastas exhibited reduced cooking time and higher cooking loss, but lower water absorption and weight of cooked pasta. The values for cooking loss and water absorption in cooked pasta suggest the need to optimize formulations in order to improve pasta quality. Color analysis revealed more reddish and yellowish tones with greater saturation. Parota flour enhances the nutritional and functional profile of the pasta, providing a healthier and more visually appealing product. These pastas enriched with parota flour show promise as functional foods by contributing to a balanced diet and encouraging the use of regional, sustainable ingredients.

## 1. Introduction

Currently, pasta is widely recognized as a dietary staple in many countries, valued for its culinary adaptability, long shelf life, low cost, and compatibility with a variety of ingredients. Despite these advantages, traditional pasta made primarily from wheat semolina has significant nutritional limitations. Such pasta typically contains high levels of carbohydrates and a moderate amount of protein but lacks essential amino acids and bioactive compounds that can contribute to human health [1]. Given these nutritional limitations of conventional pasta, there is an interest in incorporating non-traditional, nutrient-rich flours to improve the functional value of pasta. In this regard, *Enterolobium cyclocarpum* flour represents a promising ingredient due to its high protein, dietary fiber, and bioactive compounds, which could contribute to enhancing its health-promoting properties when used in cereal-based formulations. Previous studies on *Enterolobium cyclocarpum* starch have reported favorable physicochemical and functional properties, including water absorption capacity and thermal stability, suggesting its potential for application in thermally processed food systems [2]. These results support the use of parota-derived ingredients, such as seed flour, in pasta products with high nutritional value. Additionally, global dependence on wheat poses economic and environmental challenges, especially in developing countries like Mexico, where wheat production is insufficient to meet domestic demand, leading to imports and increased vulnerability to international market fluctuations [3]. Functionally beneficial foods and nutritionally balanced diets have driven research into the development of food products that not only meet basic nutritional needs but also offer additional health benefits. In this context, incorporating regional plant-based ingredients into pasta production has become a promising strategy to enhance its nutritional and functional profile. Ingredients such as legume flour, tubers, and oilseed flours have shown potential to increase the protein content, dietary fiber, and antioxidant compounds in pasta, contributing to a more balanced and healthy diet [4].

Parota flour is among the regional ingredients with the potential to enrich pasta. The parota seed, locally known as “pich”, is a legume traditionally used in animal feed but has recently gained interest due to its high nutritional content. Research suggests that parota seeds contain proteins, amino acids, and phenolic compounds with antioxidant capacity [2,5]. These nutritional attributes make parota flour an excellent candidate for use in the production of improved pasta products. However, research on the use of parota flour in food pasta is still in its early stages.

Recent studies have explored the physicochemical and functional properties of starch isolated from *Enterolobium cyclocarpum* seeds, showing their potential for food and industrial applications. Canto-Pinto et al. [6] investigated the effect of acetylation on starch obtained from parota seeds. They reported that it has favorable thermal stability, moderate amylose content, and a desirable granular morphology, supporting its suitability for modified food systems and the development of biodegradable materials. Likewise, Pérez-Pacheco et al. [7] characterized native parota starch and compared it with other unconventional starch sources, highlighting its swelling capacity, gelatinization temperature, and thermal transitions as promising properties for the development of functional foods. Despite these investigations, the use of parota flour remains largely unexplored in flour-based products such as pasta. Therefore, this study seeks to use this flour by evaluating the physicochemical quality, dietary fiber content, protein content, and antioxidant capacity in partially substituted wheat pasta formulations.

This study aims to evaluate the physicochemical quality, nutritional composition, bioactive compound content, and in vitro antioxidant capacity of pasta made with wheat semolina partially substituted with *Enterolobium cyclocarpum* (parota) flour. Specifically, pasta formulations with 10%, 30%, and 50% parota flour substitution were analyzed to determine their effect on cooking quality parameters (cooking time, cooking loss, water absorption), instrumental color analysis, and functional properties. The objective is to identify the optimal substitution level that enhances the nutritional and functional profile of the product without compromising its technological integrity or consumer acceptability. By incorporating parota flour (a regionally available and underutilized legume), this study also seeks to promote the use of local resources, contribute to dietary diversification, and reduce dependence on wheat imports through the development of innovative and health-oriented pasta products.

## 2. Materials and Methods

### 2.1. Procurement of Parota Flour (PF)

The seeds of the parota tree (*Enterolobium cyclocarpum*) were sourced from the region of Calkini, Campeche, Mexico in May 2023. The average weight of the parota pods was 20 g, each containing between 8 to 12 seeds. The seeds exhibited a color range from light to dark brown and were oval in shape, characterized by a smooth, hard seed coat. Measurements of 50 parota seeds revealed an average length of 16.5 mm, a width of 0.9 mm, and a weight of 0.8 g. The seed coats (testa) were removed by first crushing them in a grain mill, followed by manual separation. The dehulled seeds were then dried in a convection oven (Shell Lab 1350FX-10, Sheldon Manufacturing, Inc., located in Cornelius, OR, USA) at 70 °C for 72 h and subsequently stored in a desiccator to maintain dryness until the milling process. The dried seeds were processed using a commercial blender (Osterizer^®^, Boca Raton, FL, USA), followed by fine grinding in an IKA MF-10 grinder (IKA-Werke GmbH & Co., KG, Staufen, Baden-Württemberg, Germany) equipped with a 0.5 mm sieve. The ground material was then sieved through a 100-mesh screen (W.S. Tyler, Mentor, OH, USA) to produce parota seed flour (PSF). The final PSF was stored in airtight glass containers to prevent moisture absorption until further use.

The physicochemical composition of *Enterolobium cyclocarpum* (parota) flour was determined using standard AOAC methods. Moisture content was analyzed by oven drying at 105 °C until constant weight (AOAC 925.10 [8]). Ash content was determined by incineration in a muffle furnace at 550 °C (AOAC 923.03 [9]). Crude protein content was calculated using the Kjeldahl method with a nitrogen-to-protein conversion factor of 6.25 (AOAC 984.13 [10]). Crude fat was extracted by the Soxhlet method using petroleum ether as the solvent (AOAC 920.39 [11]). Total dietary fiber was determined using the enzymatic–gravimetric method involving digestion with α-amylase, protease, and amyloglucosidase (AOAC 985.29 [12]). All analyses were performed in triplicate and results were expressed on a dry weight basis.

The PSF utilized for starch extraction demonstrated the following composition: moisture content of 7.5%, ash 3.6%, crude fat 3.5%, crude fiber 2.3%, proteins 34.7%, and nitrogen-free extract 48.5%.

### 2.2. Pasta Production Process

Fettuccine pasta was prepared using durum wheat semolina and varying concentrations (10%, 30%, and 50% *w*/*w*) of parota seed flour. To avoid introducing an additional factor into the experimental design, the volume of water was kept constant in all formulations, regardless of the substitution level of durum wheat semolina. The resulting dough had an average moisture content of 32%, which meets the requirements for pasta production; therefore, no additional water was needed. Fettuccine with 0% parota flour served as control sample. The formulations consisting of durum wheat semolina, parota flour, and water were mixed in a household mixer (Kitchen Aid, Model KSM150PSOB) for 15 min. The dough was then processed using a pasta machine (Kitchen Aid, Model KPRA). The pasta was dried for 4 h at 45 °C in a convection oven (Shell Lab 1350FX-10). Two batches were prepared for each concentration.

The cooking quality of the pasta was evaluated by cooking the pasta in boiling water and the cooking time was determined when the white core of the fettuccine disappeared when compressed between two glass plates. One portion of the dried pasta was used to evaluate the cooking quality, while the other portion was cooked and then dried in a convection oven (Shell Lab 1350FX-10) at 50 °C for 48 h. Subsequently, the pasta was ground using a blender (Osterizer^®^) and stored at 4 °C in plastic containers until further analysis.

Cooked pasta samples were stored at 4 °C in sealed plastic containers for less than 12 h under strictly controlled and identical conditions for all formulations. This short storage period was selected to minimize starch retrogradation and to avoid the structural disruption associated with freezing. All analytical determinations (e.g., antioxidant capacity, phenolic content, texture analysis) were performed immediately after this brief storage interval, ensuring consistency and limiting variability across samples.

### 2.3. Proximate Chemical Composition

The proximate chemical composition of the powdered pasta was determined in duplicate according to AOAC (1997) methods for moisture (925.10), ash (923.03), protein (920.87) [13], and fat (920.39).

### 2.4. Fiber Content Determination

The fiber content was determined using the gravimetric procedure described by DeVries et al. [14]. The insoluble and soluble fiber contents were determined following the AOAC 991.42 (1995) [15] and AOAC 991.43 (1995) [16] procedures, respectively. Additionally, total fiber was determined by the procedure described in AOAC 985.29 (1995). For each determination, 0.5 g of fettuccine pasta was weighed, sieved to a size of 0.5 mm, and placed in a 125 mL Erlenmeyer flask. This procedure was performed in triplicate. To each flask, 50 mL of 0.08 M phosphate buffer at pH 6.0 was added, and the mixture was heated to 100 °C. After heating, 100 µL of thermostable α-amylase was added, and the mixture was maintained at 100 °C for 15 min, with agitation every 3 min. The samples were then allowed to cool to room temperature.

In the next stage, the pH of each sample was adjusted to 7.5 by adding 7.5 mL of 0.275 N NaOH and heating to 60 °C. Subsequently, 100 µL of a protease solution (derived from *Bacillus licheniformis*) at 50 mg/mL, prepared in 0.08 M phosphate buffer at pH 6.0, was added. The mixture was maintained at 60 °C for 30 min, with agitation every 5 min, and then allowed to cool to room temperature. In the following step, the pH of each sample was adjusted to 4.5 by adding 22 mL of 0.325 N HCl. The samples were then heated to 60 °C, and 100 µL of amyloglucosidase (derived from *Aspergillus niger*) was added. The mixtures were incubated at 60 °C for 30 min, with agitation every 5 min. After digestion, the contents of each flask were vacuum filtered. Each sample was refrigerated until further analysis.

### 2.5. Swelling Index and Water Absorption

To evaluate the swelling index (SI) of the pasta samples, a method adapted from the literature described by Cleary and Brennan [17] was used. Initially, 10 g of uncooked pasta was submerged in 100 mL of boiling distilled water and cooked to its optimal cooking time. After cooking, the pasta was drained and subsequently dried in an oven at 105 °C until a constant weight was achieved. The dried pasta was then weighed, and the swelling index was calculated using the Equation (1). The water absorption was determined according to Equation (2).

(1) $$Weight\;Increase=\frac{Weight\;of\;cooked\;pasta\;(g)}{Weight\;of\;raw\;pasta\;(g)}$$

The weight increase of pasta during cooking was determined according to AACC Method 66-50.01 [18]. This parameter was calculated as the ratio between the weight of the cooked pasta and the initial weight of the raw pasta, and it reflects the pasta’s capacity to absorb water during cooking. To maintain consistency throughout the manuscript, this parameter is referred to as weight increase, which is equivalent to what has previously been described in the literature as swelling index or water absorption ratio.

(2) $$Water\;absorption\;\left(\%\right)=\frac{cooked\;pasta\;(g)-raw\;pasta\;(g)}{raw\;pasta\;(g)}\times 100$$

### 2.6. Instrumental Color Analysis Determination

The color of the cooked fettuccine pasta was measured using a Hunter Lab MiniScan EZ colorimeter. The pasta samples were placed in the colorimeter, and the parameters L* (lightness, L* = 100 for white, L* = 0 for black), a* (+a* = red, −a* = green), and b* (+b* = yellow, −b* = blue) were recorded. The result was expressed as hue angle (Equation (3)) and chroma C* (color intensity) (Equation (4)).

(3) $$h^{*}={Tan}^{-1}\;\frac{b^{*}}{a^{*}}$$

(4) $$C^{*}=\sqrt{a^{*2}+b^{*2}}$$

### 2.7. Phenolic Compounds

The extraction of total soluble phenols was conducted according to the method described by Moo-Huchin et al. [19] with some modifications. A powdered pasta sample (2 g) was mixed with 20 mL of methanol solution (80:20 *v*/*v*), homogenized using a vortex, and sonicated for 1 h at a power of 180 W and frequency of 40 kHz. The extract was filtered through Whatman paper and stored at −20 °C until further analysis. This approach was chosen to show the actual phenolic compound content and antioxidant potential at the time of consumption, ensuring that the reported values correspond to the pasta in its final edible state.

The total soluble phenol content was determined using the method described by Singleton and Rossi [20]. In an Erlenmeyer flask, 3 mL of distilled water, 50 μL of the extract, and 250 μL of Folin-Ciocalteu reagent (1 N) were mixed. Subsequently, 750 μL of sodium carbonate solution (20%, *w*/*v*) and 950 μL of distilled water were added. After the equilibrium time was reached, the absorbance of the solution was measured at 765 nm against a solvent blank using a Perkin Elmer Lambda 11 UV-Vis spectrophotometer (PerkinElmer, Inc., Waltham, MA, USA). The concentration of total phenolic compounds was calculated using a standard curve for aqueous solutions containing gallic acid (0–10 ppm), and the result was expressed as mg gallic acid equivalents (GAE) per 100 g of dry pasta.

### 2.8. Antioxidant Capacity (ABTS Method)

The ABTS assay was performed according to the procedure described by Miller et al. [21]. The ABTS•+ cation (2,2′-azino-bis-(3-ethylbenzothiazoline-6-sulfonic acid)) was obtained by mixing 19.2 mg of ABTS•+ with 5 mL of distilled water and 88 μL of potassium persulfate (0.0378 g/mL). This solution was incubated at room temperature for 18 h. Subsequently, a 1 mL aliquot of the ABTS•+ solution was diluted in 88 μL of ethanol (96%). The radical was adjusted to an absorbance of 0.7 ± 0.2 at 734 nm. For the preparation of the reaction, 30 μL of pasta extract was mixed with 2970 μL of the adjusted ABTS•+ solution. Measurements were taken using a Perkin Elmer Lambda 11 UV-Vis spectrophotometer at 734 nm. The result was expressed as μM Trolox equivalents (TE)/100 g of sample using a calibration curve with Trolox standards at concentrations between 0 and 60 μM TE/mL.

### 2.9. Reducing Power

This method is based on the reduction of potassium ferricyanide (Fe +3) in the presence of an antioxidant, leading to the formation of a blue-colored complex K [Fe II(CN6)], which absorbs at a wavelength of 700 nm [22]. An aliquot of 2.4 mL of antioxidant extract was mixed with 2.5 mL of phosphate buffer (0.2 M, pH 6.6) and 2.5 mL of potassium ferricyanide (1% *w*/*v*, in water) in a test tube. The samples were incubated at 50 °C for 20 min in a water bath. After the incubation period, the samples were mixed with 2.5 mL of trichloroacetic acid (10% *w*/*v*, in water), shaken, and centrifuged at 3500 rpm at 25 °C for 15 min. Subsequently, a 5 mL aliquot was diluted with 5 mL of distilled water in a test tube. Then, one milliliter of ferric chloride (0.1% *w*/*v*, in water) was added and allowed to stand for 10 min at room temperature. The absorbance of the solutions was measured at 700 nm using a Perkin Elmer Lambda 11 UV-Vis spectrophotometer. The result was expressed as mg vitamin C equivalents/100 g of edible portion using a calibration curve with vitamin C standards at concentrations between 5 and 30 ppm.

### 2.10. Statistical Analysis

Statistical analysis was conducted using Statgraphics Plus, version 5.1. Each sample was analyzed in duplicate. Results were expressed as mean ± standard deviation. Data were evaluated using one-way analysis of variance (ANOVA), and means were compared using Tukey’s test (*p* < 0.05).

## 3. Results and Discussion

### 3.1. Proximte Chemical Composition of Cooked Pastas

The proximate analysis of pasta with varying levels of parota flour (0–50%) showed nutritional differences, while moisture content remained stable across all formulations (5.24–5.97%) (Table 1). Since all pasta samples were prepared with a standardized dough moisture of 32% and dried under identical conditions (temperature, duration, and air flow), the observed differences are minimal and statistically non-significant. These results confirm the drying process was effectively standardized, and flour substitution had no substantial impact on the final moisture content of the dried pasta [23]. During mixing and kneading, both flours absorb water effectively, ensuring uniform moisture in the dough. The water amount is precisely adjusted to achieve optimal consistency, forming a cohesive and elastic dough. This careful calibration maintains proper hydration, resulting in high-quality pasta regardless of the flour type used [24]. Although unconventional flours may exhibit different water absorption behaviors during dough formation, the final moisture content of the dried pasta samples remained relatively stable, likely due to the standardized drying conditions applied. Therefore, the minor differences observed can be attributed more to the drying process than to the flour composition itself. The slight variations observed in the moisture content of the dried pasta samples are primarily attributed to the drying conditions rather than the composition of the dough itself. In dried pasta, final moisture levels are predominantly influenced by factors such as drying temperature, air flow, and duration, which determine the rate of moisture loss and the attainment of equilibrium. Although the composition of the dough can affect water retention and drying kinetics, its influence is secondary when compared to the impact of thermal parameters. The low degree of variability across samples indicates that the drying process was well-standardized, ensuring consistent moisture content irrespective of the formulation used.

Additionally, the drying stage plays a critical role in minimizing initial differences in water retention associated with different flour types. Following lamination, the pasta was subjected to a controlled drying protocol designed to reduce moisture to stable levels for product preservation. This procedure effectively minimized the influence of flour-specific hydration behavior on the final moisture content [25]. Although the nutritional composition of *Enterolobium cyclocarpum* flour differs from that of wheat semolina, its physical properties (such as particle size and fiber content) exhibit comparable water interaction characteristics. The presence of starch and dietary fiber in parota flour contributes similarly to water absorption during drying, resulting in moisture contents that are consistent with those of conventional formulations [26].

The ash content is a direct indicator of the mineral quantity present in food, which is crucial for assessing its nutritional contribution. In this study, a significant increase in ash content was observed with the incorporation of parota flour, being highest in pastas with 50% parota flour (1.25%) compared to the control pasta (0.76%). The inclusion of parota flour in the pastas enhances the total mineral content and diversifies the mineral profile, providing essential nutrients vital for numerous biological functions, including bone formation, muscle contraction, and regulation of fluid and acid-base balance in the body [27]. Although an increase in ash content was observed in pasta formulations enriched with *Enterolobium cyclocarpum* flour, it is important to note that this measurement only reflects total mineral content and does not consider bioavailability. Legume-based flours, including parota flour, are known to contain phytic acid, an antinutritional factor that can chelate divalent minerals such as iron, zinc, and calcium, reducing their intestinal absorption. Consequently, the actual nutritional contribution of these minerals may be significantly lower than their total content suggests. This limitation is particularly relevant for plant-based formulations, where phytates may be present in substantial amounts.

The variation in ash content among the formulations can be explained by the different mineral concentrations in the flours used. Parota flour, derived from a legume, has a distinct mineral profile compared to wheat semolina, which is more limited in certain micronutrients. This result is consistent with other studies that have demonstrated that the addition of legume and other vegetable flours can significantly enrich the mineral content of cereal products [28]. The observed increase in ash content in pasta formulations enriched with parota flour suggests a possible increase in the presence of minerals but does not confirm it. Therefore, additional specific analyses are required and are the subject of future studies.

The increase in ash content in pastas enriched with parota flour also confers practical and sensory implications. From a practical standpoint, products with higher mineral content can offer additional health benefits, especially in diets where micronutrients are limited. However, it is crucial to ensure that this enrichment does not negatively impact the sensory properties of the product, such as taste and texture [24].

The results regarding the fat content in pastas with different concentrations of parota flour show significant changes, which have important implications for the nutritional profile and functional quality of the products. A progressive increase in fat content is observed as the proportion of parota flour in the formulations increases. The control pasta (PS0) has the lowest fat content (0.44%), while the pasta with 50% parota flour (PP50) shows the highest value (1.34%). The lipid content of the pasta formulations increased slightly with the addition of *Enterolobium cyclocarpum* flour, ranging from 1.8% to 3.2% (dry weight basis), depending on the substitution level. Despite this increase, the total fat content remains within the typical range for cereal-based enriched products and is not considered excessive. According to standard dietary guidelines, a typical serving of dry pasta (70–100 g) would provide a moderate amount of fat, within the daily intake limits recommended by the World Health Organization [29]. Furthermore, consumption of pasta enriched with parota flour is unlikely to pose any health risks in terms of lipid intake when consumed as part of a balanced diet. This increase in fat content can be directly attributed to the composition of parota seeds, which contain beneficial lipids, including essential unsaturated fatty acids such as linoleic and oleic acids [30]. The incorporation of parota flour not only increases the total fat content in the pastas but also enhances the quality of the fatty acid profile, which can have positive effects on cardiovascular health and inflammation reduction [31]. The variability in fat content among the different formulations can be explained by the differential concentration of lipids in the flours used. Parota flour contributes a significant amount of lipids compared to wheat semolina, resulting in a proportional increase in fat content in the pastas as the proportion of parota flour increases. These results are consistent with other research that reports that the addition of legume and other vegetable flours can enrich the lipid content of cereal products [32]. The fat content of the pasta progressively increased with the addition of *Enterolobium cyclocarpum* flour, confirming its higher intrinsic lipid content compared to wheat semolina. The value obtained was 0.44% for the control pasta (PS0), 0.57% for PP10, 0.91% for PP30, and 1.34% for PP50 (dry weight). Lipids were not identified because a fatty acid profile was not performed.

Additionally, the presence of lipids in the flours can influence the texture and palatability of the pastas. Lipids can act as plasticizers, improving the workability of the dough and contributing to a softer and more acceptable texture in the final product. This aspect is crucial for maintaining the sensory acceptability of enriched pastas, ensuring that the increase in fat content does not negatively affect the organoleptic properties of the product [24].

From a nutritional perspective, the increase in fat content also exhibits practical advantages. The unsaturated fatty acids present in parota flour are known for their beneficial effects on health, including the improvement of blood lipid profiles and the reduction of the risk of chronic diseases such as atherosclerosis and type 2 diabetes [33]. Therefore, pastas enriched with parota flour can contribute to a more balanced diet.

The results for protein content showed that the control pasta (PS0) contains 13.41% protein, while the pasta with 50% parota flour (PP50) reaches 22.06%. This substantial increase in protein content is due to the high protein concentration of parota flour, which contains significant amounts of essential amino acids such as lysine, leucine, isoleucine, and threonine, which are crucial for protein synthesis and maintaining health [2]. The inclusion of parota flour significantly enhances the amino acid profile of the pastas, overcoming the limitation of essential amino acids commonly found in traditional pastas made exclusively with wheat semolina [23]. This protein content enrichment increases the total protein amount and improves the protein quality of the product, providing a more complete balance of essential amino acids necessary for growth and tissue repair [34]. The increase in protein content can positively influence the functional properties of the pastas. Proteins play a critical role in the development of pasta structure, enhancing its elasticity and firmness. The addition of parota flour can improve these mechanical properties, resulting in pasta with better texture and higher cooking resistance, which is essential for maintaining the sensory quality of the final product [27]. Moreover, plant proteins have been shown to have beneficial health effects. Recent studies have associated the consumption of plant proteins with a reduced risk of cardiovascular diseases, better blood glucose control, and a lower incidence of certain types of cancer [35]. This is partly due to the presence of bioactive compounds in plant proteins that can act as antioxidants and anti-inflammatory agents [36].

Parota flour, being a source of plant-based proteins, also contributes to food sustainability. The production of plant proteins generally requires fewer natural resources and emits fewer greenhouse gases compared to animal proteins, making pastas enriched with parota flour a more sustainable and eco-friendly option [37].

### 3.2. Dietary Fiber Content

The analysis of dietary fiber content (see Table 2) in pasta with varying percentages of parota flour substitution shows significant changes. The control pasta (PS0) exhibits a total dietary fiber content of 9.5 g/100 g, with 1.12 g/100 g of soluble fiber and 8.42 g/100 g of insoluble fiber. Upon incorporating parota flour, there is a notable increase in total dietary fiber content, reaching 22.1 g/100 g in the pasta with 50% parota flour (PP50). This increase is evident in both soluble and insoluble fiber, though differentially based on the level of substitution.

Dietary fiber is essential for digestive and metabolic health. Soluble fibers, such as those found in pasta with parota flour, have demonstrated positive effects on blood sugar regulation and reduction of LDL cholesterol [34]. The pasta with 10% parota flour (PP10) shows a significantly higher soluble fiber content (4.04 g/100 g) compared to other formulations. This suggests that even a low incorporation of parota flour can substantially improve the soluble fiber profile of pasta, offering potential benefits for cardiovascular health and glycemic control [27]. In contrast, insoluble fiber, which is predominant in pasta enriched with parota flour, is essential for digestive health. It facilitates intestinal transit and prevents constipation while contributing to a feeling of satiety, which can be beneficial for weight management [28]. In pasta with 50% parota flour (PP50), the insoluble fiber content reaches 20.18 g/100 g, a significant increase compared to the control pasta. This rise can be attributed to the high concentration, making these pastas a suitable option for enhancing dietary fiber intake.

The variability in total dietary fiber content and its fractions (soluble and insoluble) among the different formulations can be explained by the intrinsic composition of parota flour. Parota seeds are rich in dietary fibers, which accounts for the proportional increase in pasta as the substitution percentage of the flour increases [35]. These results align with previous studies that have documented the significant improvement in dietary fiber content in food products through the incorporation of legume and other vegetable flours [36]. The increase in dietary fiber content by incorporating *Enterolobium cyclocarpum* flour, especially at higher substitution levels, had a notable impact on the technological quality of pasta. Dietary fibers, especially insoluble fibers, can interfere with the formation and continuity of the gluten network by physically disrupting protein-starch interactions. Furthermore, fibers compete for water during dough hydration, reducing its availability for gluten development and starch gelatinization. As a result, high-fiber pasta formulations exhibit lower water absorption, lower cooked weight, and higher cooking loss. These effects have been reported in previous studies involving the addition of high-fiber ingredients to wheat-based pasta [38,39].

### 3.3. Color Determination

Table 3 presents the color results for the pasta samples, providing a comprehensive understanding of how the incorporation of parota flour affects the color of the pasta.

Lightness (L*) is a parameter that indicates how light or dark a sample is. The L* values show that the control pasta (PS0) possesses a value of 80.42, while the pasta with the highest percentage of parota flour (PP50) displays a slightly lower value of 79.61. Although this difference is not statistically significant, it suggests a slight decrease in lightness with increased parota flour. This may be attributed to the natural pigments present in parota flour, which can influence the final color of the product [34].

The a* parameter indicates color ranging from red (+a*) to green (−a*). It is observed that the pasta with parota flour (PP10, PP30, and PP50) has slightly higher a* values compared to the control pasta. PP50 has a value of 1.26 compared to 0.79 for PS0. This increase in a* value indicates a trend towards more reddish tones, which is consistent with the presence of phenolic compounds and other pigments in parota flour [27]. The variation in a* value is significant and can influence the visual appearance and the perception of naturalness and healthiness of the product.

Parameter b*, which indicates the color position between yellow and blue, also shows a significant increase with the addition of parota flour. The control pasta has a b* value of 25.62, while PP50 reaches a value of 31.28. This increase suggests a trend towards more yellow tones, potentially enhancing consumer appeal, as brighter and warmer colors are generally preferred in food products [28].

Hue angle and chroma (C*) are parameters that describe the purity and intensity of the color. The hue angle shows minor variations among the different formulations, with values ranging from 87.68 to 88.58. Although these differences are small, they indicate that the color hue remains relatively constant, but with a slight trend towards warmer tones in the pasta with higher parota flour content. Chroma, on the other hand, shows a significant increase with the addition of parota flour, especially in PP50, which has a value of 31.31 compared to 25.63 for PS0. This suggests that pasta with parota flour is not only brighter but also has a more saturated and vivid color [35].

Incorporating parota flour into pasta significantly affects its color properties, making the pasta slightly darker, with more reddish and yellowish tones, and greater color saturation. These changes can enhance the visual appeal of the product, making it more attractive to consumers who seek foods with natural ingredients and additional health benefits. These results underscore the importance of considering color attributes when developing new pasta formulations, as color is a key factor in consumer acceptance and product quality perception.

### 3.4. Cooking Quality

Table 4 presents the results of the cooking quality of the pasta samples. Cooking time is a critical indicator of product efficiency and convenience for consumers. It was observed that pasta with parota flour (PP10, PP30, PP50) required less cooking time (8 min) compared to the control pasta (PS0), which needed 11 min. This reduction in cooking time can be attributed to the presence of compounds in parota flour that facilitate faster starch gelatinization and more efficient protein denaturation during cooking [35]. A shorter cooking time enhances consumer convenience and can reduce energy consumption during preparation.

Cooking loss, which measures the amount of solutes released into the water during cooking, is an important parameter for evaluating the structural integrity of the pasta. Pastas with 30% and 50% parota flour (PP30 and PP50) showed a higher cooking loss (6%) compared to the control pasta and pasta with 10% parota flour (PP10), which had a cooking loss of 5%. This increase may be due to a less robust gluten network in pastas with higher parota flour content, allowing greater leaching of solutes during cooking [27]. However, it is important to note that these cooking loss values are within acceptable limits for high-quality pasta [40]. The proteins present in *Enterolobium cyclocarpum* flour are of plant origin and lack the viscoelastic properties characteristic of gluten-forming proteins. Unlike wheat gliadins and glutenins, which interact to form a continuous, extensible, and elastic three-dimensional gluten network, parota proteins do not contribute to this structural matrix. The partial substitution of semolina with parota flour likely diluted the gluten network, compromising its integrity and reducing its ability to retain water and starch during cooking. This may account for the increased cooking losses and decreased water absorption in the enriched pasta samples. Additionally, the weakening of protein–starch interactions could have resulted in a less cohesive structure, negatively impacting firmness and elasticity. Therefore, while parota flour enhances nutritional and functional profiles, its influence on dough rheology and pasta structure highlights the need to optimize substitution levels and consider protein complementation or structural stabilizers to preserve desirable textural qualities.

The weight of cooked pasta is another quality indicator, reflecting the pasta’s ability to absorb water during cooking. The control pasta (PS0) showed the highest weight of cooked pasta (26 g/100 g), while pasta with 50% parota flour (PP50) had the lowest weight (21.3 g/100 g). This decrease in the weight of cooked pasta with increasing parota flour content can be attributed to a lower starch content and a higher concentration of proteins and fiber, which have a lower water absorption capacity [41]. The water-holding capacity of the flour components and the more compact structure associated with increased protein levels likely contributed to the reduction in cooked pasta.

The swelling index and water absorption are interrelated parameters indicating the pasta’s ability to absorb and retain water, which is crucial for its final texture. The control pasta had the highest swelling index (1.92) and water absorption (160%), while pasta with 50% parota flour showed the lowest values (1.63 and 113%, respectively). These results suggest that the inclusion of parota flour reduces the pasta’s ability to swell and absorb water, potentially resulting in a firmer and less sticky texture [42].

The incorporation of parota flour in pasta formulations significantly affects cooking quality. While it reduces cooking time and improves certain aspects of texture, it also increases cooking loss and decreases water absorption and the weight of cooked pasta. These results are consistent with those reported in the literature by Qin et al. [43], suggesting that flours from unconventional sources can alter the structural and functional properties of pasta. These findings underscore the importance of balancing ingredient proportions to optimize the nutritional quality and cooking characteristics of enriched pasta.

### 3.5. Phenolic Compounds, Antioxidant Capacity, and Reducing Power

Table 5 presents the results for total phenolic compounds (TPC) content, antioxidant capacity, and reducing power. TPC are known for their antioxidant properties, which can significantly contribute to the prevention of chronic diseases by counteracting the harmful effects of free radicals [34]. The results of the TPC show that the control pasta (PS0) does not contain detectable amounts of these compounds, while pastas with parota flour exhibit significant increases in TPC content depending on the substitution percentage. The pasta with 10% parota flour (PP10) has a phenolic content of 1.58 mg/100 g of gallic acid equivalents, which increases to 7.31 mg/100 g in the pasta with 30% parota flour (PP30) and reaches 23.35 mg/100 g in the pasta with 50% parota flour (PP50). This considerable increase is due to the high concentration of TPC present in parota seeds, which are transferred to the final product during pasta production [27]. The TPC content in the pasta with 50% parota flour was 23.35 mg/100 g gallic acid equivalents, while no TPC was detected in the control pasta. These values are higher than those reported in the literature for pastas enriched with other plant ingredients. Schmidt et al. [44] reported that lentil-enriched pasta contained approximately 15 mg of TPC per 100 g. In comparison, the higher TPC content observed in parota flour-enriched pasta in our study suggests that *Enterolobium cyclocarpum* flour may be a more concentrated source of antioxidant compounds than many commonly used legume flours.

The antioxidant capacity, measured by the ABTS assay, also shows a significant increase with the addition of parota flour. While no antioxidant capacity was detected in the control pasta, the pasta with 10% parota flour (PP10) shows an activity of 16.54 µM/100 g Trolox equivalents. This value increases to 51.59 µM/100 g in the pasta with 30% parota flour and reaches 61.78 µM/100 g in the pasta with 50% parota flour. These results indicate that parota flour enhances the phenolic content and boosts the antioxidant capacity of the pasta, which is crucial for its functionality and potential health impact.

The reducing power, another important indicator of antioxidant capacity, shows a similar pattern. The control pasta exhibited a reducing power of 5.81 mg vitamin C equivalents per 100 g, whereas the pasta enriched with 10% parota flour (PP10) showed an increased value of 17.93 mg/100 g. The pasta with 30% parota flour (PP30) reaches a reducing power of 25.61 mg/100 g, and the pasta with 50% parota flour (PP50) shows a value of 40.19 mg/100 g. This increase in reducing power is consistent with the increase in TPC content and antioxidant capacity, highlighting the synergy between these bioactive components [28]. Notably, La Gatta et al. [45] reported values of 30 mg/100 g in quinoa flour-enriched pasta, which is lower than the values obtained in this study for parota flour-enriched pasta. These results underscore the effectiveness of parota flour in enhancing the antioxidant properties of pasta, likely due to the synergy between the different TPC present.

These results have important implications for the food industry and public health. Incorporating parota flour into pasta formulations enriches its nutritional content and confers improved functional properties, enhancing its value as a functional food. TPC and antioxidant capacity are known for their role in reducing the risk of cardiovascular diseases, cancer, and other chronic diseases, making these enriched pastas an attractive option for health-conscious consumers [36].

The incorporation of parota flour into pasta formulations significantly increases the TPC content, antioxidant capacity, and reducing power of the final product. These changes enhance the nutritional and functional value of pasta, offering potential health benefits.

## 4. Conclusions

In this study, physicochemical quality, bioactive compound content, and antioxidant capacity of pasta made with different percentages of parota flour (*Enterolobium cyclocarpum*) substitution were evaluated. The results indicate that incorporating parota flour significantly improves the nutritional and functional profile of the pasta.

The parota flour-enriched pasta showed a notable increase in protein content, rising from 13.41% in the control pasta to 22.06% in the pasta with 50% substitution. This increase in protein content is accompanied by an improvement in the essential amino acid profile, which is crucial for protein synthesis and health maintenance. Additionally, the incorporation of parota flour also elevated the total dietary fiber content, reaching 22.1 g/100 g in the pasta with 50% substitution, compared to 9.5 g/100 g in the control pasta. This improvement is evident in both soluble and insoluble fiber, offering additional benefits for digestive and metabolic health.

Among the formulations evaluated, pasta with 30% parota flour was identified as the optimal substitution level, as it provides a balance between nutritional enhancement and technological performance. At this level, significant improvements were achieved in protein content, dietary fiber, and antioxidant capacity, while cooking quality remained within acceptable limits.

Color analysis revealed that the parota flour-enriched pasta exhibited slight changes towards more reddish and yellowish tones, with greater color saturation, which can enhance the visual appeal of the product. In terms of cooking quality, the parota flour-enriched pasta showed a reduced cooking time and increased cooking loss but lower water absorption and weight of cooked pasta. The reduction in optimal cooking time observed in pasta enriched with parota flour could be associated with structural modifications in the pasta matrix due to gluten dilution and the incorporation of fiber-rich components. These changes suggest a firmer and less sticky texture.

The total phenolic compounds, antioxidant capacity, and reducing power also increased significantly with the incorporation of parota flour. The pasta with 50% parota flour showed a phenolic compound content of 23.35 mg/100 g, an antioxidant capacity of 61.78 µM/100 g Trolox equivalents, and a reducing power of 40.19 mg/100 g vitamin C equivalents. The results of this study highlight the potential of *Enterolobium cyclocarpum* flour as a valuable ingredient for the development of nutritionally and functionally enriched pastas. The incorporation of this underutilized legume could contribute to improving the nutritional quality of foods and support the diversification of food resources, promote the use of local crops, and align with sustainable food development goals.

## Figures and Tables

**Table 1:** Proximate chemical composition of cooked pasta added with parota seed flour.

Sample	Moisture (%)	Ashes (%)	Fat (%)	Protein (%)
PS0	5.24 ± 0.01 a	0.76 ± 0.01 b	0.44 ± 0.01 a	13.41 ± 0.18 a
PP10	5.97 ± 0.01 a	0.40 ± 0.01 a	0.57 ± 0.03 a	17.01 ± 0.01 b
PP30	5.41 ± 0.01 a	0.81 ± 0.01 b	0.91± 0.05 b	19.38 ± 0.61 c
PP50	5.75 ± 0.03 a	1.25 ± 0.06 c	1.34 ± 0.08 c	22.06 ± 0.28 d

Values are mean ± standard deviation (*n* = 3), dry basis. Different letters in the same column indicate a significant statistical difference (*p* < 0.05). PS0, denotes 0% added parota flour; PP10, denotes 10% added parota flour; PP30, denotes 30% added parota flour; PP50, denotes 50% added parota flour.

**Table 2:** Dietary fiber content.

Sample	Total Dietary Fiber (g/100 g)	Soluble Fiber (g/100 g)	Insoluble Fiber (g/100 g)
PS0	9.5 ± 0.1 a	1.12 ± 0.1 a	8.42 ± 0.1 a
PP10	15.8 ± 0.1 b	4.04 ± 0.1 d	11.84 ± 0.1 b
PP30	19.4 ± 0.1 c	2.14 ± 0.1 c	17.26 ± 0.1 c
PP50	22.1 ± 0.1 d	1.94 ± 0.1 b	20.18 ± 0.1 d

Values are mean ± standard deviation (*n* = 3), dry basis. Different letters in the same column indicate a significant statistical difference (*p* < 0.05). PS0, denotes 0% added parota flour; PP10, denotes 10% added parota flour; PP30, denotes 30% added parota flour; PP50, denotes 50% added parota flour.

**Table 3:** Color parameters of pasta added with parota seed flour.

Sample	L*	a*	b*	Tone (°hue)	Chromaticity (C*)
PS0	80.42 ± 0.31 a	0.79 ± 0.04 a	25.62 ± 0.23 a	88.22 ± 0.09 b	25.63 ± 0.23 a
PP10	80.36 ± 0.11 a	1.17 ± 0.05 b	30.40 ± 0.52 b	87.79 ± 0.06 a	30.42 ± 0.52 b
PP30	80.30 ± 0.08 a	0.75 ± 0.04 a	30.30 ± 0.31 b	88.58 ± 0.07 c	30.31 ± 0.31 b
PP50	79.61 ± 0.39 a	1.26 ± 0.01 b	31.28 ± 0.15 b	87.68 ± 0.02 a	31.31± 0.15 b

Values are mean ± standard deviation (*n* = 3), dry basis. Different letters in the same column indicate a significant statistical difference (*p* < 0.05). PS0, denotes 0% added parota flour; PP10, denotes 10% added parota flour; PP30, denotes 30% added parota flour; PP50, denotes 50% added parota flour.

**Table 4:** Cooking quality of pasta added with parota seed flour.

Fettuccine Pasta	Cooking Time (min)	Cooking Loss (%)	Pasta Weight Cooked (g/100 g)	Weight Increase (g/g)	Water Absorption (%)
PS0	11 ± 0.1 b	5 ± 0.1 a	26 ± 0.1 d	1.92 ± 0.1 d	160 ± 0.3 d
PP10	8 ± 0.1 a	5 ± 0.2 a	24.3 ± 0.1 c	1.89 ± 0.1 c	143 ± 0.5 c
PP30	8 ± 0.1 a	6 ± 0.1 b	23 ± 0.1 b	1.61 ± 0.1 a	130 ± 0.4 b
PP50	8 ± 0.1 a	6 ± 0.1 b	21.3± 0.1 a	1.63 ± 0.1 b	113 ± 0.3 a

Values are mean ± standard deviation (*n* = 3), dry basis. Different letters in the same column indicate a significant statistical difference (*p* < 0.05). PS0, denotes 0% added parota flour; PP10, denotes 10% added parota flour; PP30, denotes 30% added parota flour; PP50, denotes 50% added parota flour.

**Table 5:** Content of total phenolic compounds and the antioxidant capacity of pastas added with parota seed flour.

	Equivalent to Gallic Acid (mg/100 g)	Equivalent to Trolox (µM/100 g) ABTS	Equivalent to Vitamin C (mg/100 g)
Sample	Phenolic Compounds		Reducing Power
PS0	Not detected	Not detected	5.81 ± 0.13 a
PP10	1.58 ± 0.01 a	16.54 ± 4.24 a	17.93 ± 0.23 b
PP30	7.31 ± 1.87 b	51.59 ± 1.43 b	25.61. ± 1.15 c
PP50	23.35 ± 1.24 c	61.78 ± 1.43 c	40.19 ± 2.08 d

Values are mean ± standard deviation (*n* = 3), dry basis. Different letters in the same column indicate a significant statistical difference (*p* < 0.05). PS0, denotes 0% added parota flour; PP10, denotes 10% added parota flour; PP30, denotes 30% added parota flour; PP50, denotes 50% added parota flour.

## Data Availability

The original contributions presented in the study are included in the article, further inquiries can be directed to the corresponding author.

## References

[1] Wang J., Brennan M.A., Serventi L., Brennan C.S. (2022). Impact of functional vegetable ingredients on the technical and nutritional quality of pasta. Crit. Rev. Food Sci. Nutr..

[2] Estrada-León R.J., Moo-Huchin V.M., Ríos-Soberanis C.R., Betancur-Ancona D., May-Hernández L.H., Carrillo-Sánchez F.A., Cervantes-Uc J.M., Pérez-Pacheco E. (2016). The effect of isolation method on properties of parota (*Enterolobium cyclocarpum*) starch. Food Hydrocoll..

[3] Zavala Álvarez J., Martínez Partida J.A., Gastélum López L.d.C. (2022). La organización de productores de trigo de Baja California. Polis.

[4] Ainsa A., Roldan S., Marquina P.L., Roncalés P., Beltrán J.A., Calanche Morales J.B. (2022). Quality parameters and technological properties of pasta enriched with a fish by-product: A healthy novel food. J. Food Process. Preserv..

[5] Ekanem N., Inyang U., Ikwunze K. (2022). Chemical composition, secondary metabolites and nutritive value of elephant-ear tree (*Enterolobium cyclocarpum* (Jacq.) Griseb): A review. Niger. J. Anim. Prod..

[6] Canto-Pinto J.C., Pérez-Pacheco E., Ríos-Soberanis C.R., Ortiz-Fernández A., Estrada-León R.J., Moo-Huchin V.M., Pérez-Padilla Y. (2024). Effects of Acetylation on the Morphological, Physicochemical, and Thermal Properties of *Enterolobium cyclocarpum* Starch. ChemistrySelect.

[7] Pérez-Pacheco E., Ortiz-Fernández A., Ríos-Soberanis C., Estrada-León R., Moo-Huchín V., Pérez-Padilla Y., Canto-Pinto J.C., Dzul-Cervantes M.A. (2024). Characterization of Unconventional Sources of Starch: Physicochemical and Thermal Properties. Res. Sq..

[8] (2019). Moisture in Flour. Official Methods of Analysis of AOAC International.

[9] (2019). Ash in Flour. Official Methods of Analysis of AOAC International.

[10] (2019). Crude Protein in Animal Feed, Forage (Alternative II), Grain and Oilseed Products. Official Methods of Analysis of AOAC International.

[11] (2019). Fat (Crude) in Flour. Official Methods of Analysis of AOAC International.

[12] (2019). Total Dietary Fiber in Foods—Enzymatic-Gravimetric Method. Official Methods of Analysis of AOAC International.

[13] (2019). Protein (Crude) in Flour. Official Methods of Analysis of AOAC International.

[14] DeVries J.W., Rader J.I. (2005). Historical perspective as a guide for identifying and developing applicable methods for dietary fiber. J. AOAC Int..

[15] (2019). Insoluble Dietary Fiber in Foods—Enzymatic-Gravimetric Method. Official Methods of Analysis of AOAC International.

[16] (2019). Total, Soluble, and Insoluble Dietary Fiber in Foods—Enzymatic-Gravimetric Method. Official Methods of Analysis of AOAC International.

[17] Cleary L., Brennan C. (2006). The influence of a (1→3)(1→4)-*β*-d-glucan rich fraction from barley on the physico-chemical properties and in vitro reducing sugars release of durum wheat pasta. Int. J. Food Sci. Technol..

[18] (2010). Pasta and Noodle Cooking Quality—Firmness. Approved Methods of Analysis.

[19] Moo-Huchin V.M., Estrada-Mota I., Estrada-León R., Cuevas-Glory L., Ortiz-Vázquez E., Vargas M.d.L.V.y., Betancur-Ancona D., Sauri-Duch E. (2014). Determination of some physicochemical characteristics, bioactive compounds and antioxidant activity of tropical fruits from Yucatan, Mexico. Food Chem..

[20] Singleton V.L., Rossi J.A. (1965). Colorimetry of Total Phenolics with Phosphomolybdic-Phosphotungstic Acid Reagents. Am. J. Enol. Vitic..

[21] Miller N.J., Rice-Evans C., Davies M.J. (1993). A new method for measuring antioxidant activity. Biochem. Soc. Trans..

[22] Yen G.-C., Chen H.-Y. (1995). Antioxidant Activity of Various Tea Extracts in Relation to Their Antimutagenicity. J. Agric. Food Chem..

[23] Romano A., Ferranti P., Gallo V., Masi P. (2021). New ingredients and alternatives to durum wheat semolina for a high quality dried pasta. Curr. Opin. Food Sci..

[24] Sissons M. (2022). Development of Novel Pasta Products with Evidence Based Impacts on Health—A Review. Foods.

[25] Bresciani A., Pagani M.A., Marti A. (2022). Pasta-Making Process: A Narrative Review on the Relation between Process Variables and Pasta Quality. Foods.

[26] Serratos Arévalo J.C., Carreón Amaya J., Castañeda Vázquez H., Garzón De la Mora P., García Estrada J. (2008). Composición químico-nutricional y de factores antinutricionales en semillas de parota (*enterolobium cyclocarpum*). Interciencia.

[27] Amri Z., Bhouri A.M., Dhibi M., Hammami M., Hammami S., Mechri B. (2024). Nutritional composition, lipid profile and stability, antioxidant activities and sensory evaluation of pasta enriched by linseed flour and linseed oil. BMC Biotechnol..

[28] Trigo J.P., Alexandre E.M.C., Saraiva J.A., Pintado M.E. (2022). High value-added compounds from fruit and vegetable by-products—Characterization, bioactivities, and application in the development of novel food products. Crit. Rev. Food Sci. Nutr..

[29] WHO Healthy Diet: Key Facts. https://www.who.int/es/news-room/fact-sheets/detail/healthy-diet.

[30] Demir B., Bilgiçli N. (2021). Utilization of quinoa flour (Chenopodium quinoa Willd.) in gluten-free pasta formulation: Effects on nutritional and sensory properties. Food Sci. Technol. Int..

[31] Messia M.C., Cuomo F., Falasca L., Trivisonno M.C., De Arcangelis E., Marconi E. (2021). Nutritional and Technological Quality of High Protein Pasta. Foods.

[32] Siegrist M., Hartmann C. (2020). Consumer acceptance of novel food technologies. Nat. Food.

[33] Demir B., Bilgiçli N. (2020). Changes in chemical and anti-nutritional properties of pasta enriched with raw and germinated quinoa (*Chenopodium quinoa* Willd.) flours. J. Food Sci. Technol..

[34] Gazza L., Nocente F. (2022). Innovative Pasta with High Nutritional and Health Potential. Foods.

[35] Barber T.M., Kabisch S., Pfeiffer A.F.H., Weickert M.O. (2020). The Health Benefits of Dietary Fibre. Nutrients.

[36] Ferrari L., Panaite S.-A., Bertazzo A., Visioli F. (2022). Animal- and Plant-Based Protein Sources: A Scoping Review of Human Health Outcomes and Environmental Impact. Nutrients.

[37] Detzel A., Krüger M., Busch M., Blanco-Gutiérrez I., Varela C., Manners R., Bez J., Zannini E. (2022). Life cycle assessment of animal-based foods and plant-based protein-rich alternatives: An environmental perspective. J. Sci. Food Agric..

[38] Bouasla A., Wójtowicz A., Zidoune M.N. (2017). Gluten-free precooked rice pasta enriched with legumes flours: Physical properties, texture, sensory attributes and microstructure. LWT.

[39] Hassan O.M.S., Di Folco U., Nardone M.R., Tubili F., Tubili C. (2020). Fiber enrichment of pasta: Metabolic effects and diet adherence in obese subjects. Mediterr. J. Nutr. Metab..

[40] Ortolan F., Steel C.J. (2017). Protein Characteristics that Affect the Quality of Vital Wheat Gluten to be Used in Baking: A Review. Compr. Rev. Food Sci. Food Saf..

[41] Cimini A., Cibelli M., Taddei A.R., Moresi M. (2021). Effect of cooking temperature on cooked pasta quality and sustainability. J. Sci. Food Agric..

[42] Ainsa A., Honrado A., Marquina P.L., Roncalés P., Beltrán J.A., Calanche M.J.B. (2021). Innovative Development of Pasta with the Addition of Fish By-Products from Two Species. Foods.

[43] Qin P., Wang T., Luo Y. (2022). A review on plant-based proteins from soybean: Health benefits and soy product development. J. Agric. Food Res..

[44] Schmidt H.d.O., Oliveira V.R.d. (2023). Overview of the Incorporation of Legumes into New Food Options: An Approach on Versatility, Nutritional, Technological, and Sensory Quality. Foods.

[45] la Gatta B., Rutigliano M., Liberatore M.T., Dilucia F., Spadaccino G., Quinto M., Di Luccia A. (2023). Preservation of bioactive compounds occurring in fresh pasta fortified with artichoke bracts and tomato powders obtained with a novel pre-treatment. LWT.

